# Percutaneous Intervention of LVAD Outflow Graft Obstruction and Thrombosis

**DOI:** 10.14797/mdcvj.1360

**Published:** 2024-04-04

**Authors:** Andrew Takla, Fahad Eid, Mostafa Elbanna, Mohamed Magdi Eid, Akshay Joshi, Abdallah Bitar, Ryan Lydon, Scott Feitell

**Affiliations:** 1 Rochester General Hospital, Rochester, New York, US; 2 Unity Hospital, Rochester, New York, US

**Keywords:** left ventricular assist device outflow graft thrombosis, LVAD, graft stenting, pump exchange

## Abstract

Left ventricular assist devices serve as a salvage therapy for patients with advanced heart failure. Complications such as thrombosis and obstruction can lead to acute device malfunction, posing significant clinical risks. A multidisciplinary approach is crucial for management. Few cases in the literature have demonstrated the safety and efficacy of percutaneous intervention, which holds significant value due to its less invasive nature and minimal risk of morbidity, especially in high-risk surgical patients.

## Introduction

Left ventricular assist devices (LVADs) represent a pivotal advancement in the treatment of patients with advanced heart failure, particularly for those awaiting transplantation or for whom transplantation is not an option. By offering enhanced circulatory support, these devices can dramatically improve both survival and quality of life. However, as with any intricate medical intervention, LVADs come with their own set of challenges and complications. Thrombosis and obstruction stand out due to their potential to precipitate acute device malfunction, leading to significant clinical instability. These complications necessitate a rigorous and multidisciplinary approach for both diagnosis and management. This case report delves into the management of LVAD obstruction, emphasizing the importance of coordinated care. By combining the expertise of heart failure specialists, interventional cardiologists, and vascular and cardiac surgeons, we illustrate the path to optimal patient outcomes in the face of such a complex clinical scenario.

## Case Presentation

A 68-year-old male with a medical history of ischemic cardiomyopathy has a status of post-coronary artery bypass graft, mitral valve replacement, and HeartMate III (Abbott Cardiovascular) left ventricular assist device implantation as a bridge to transplant in 2020. He is on coumadin, has immune thrombocytopenic purpura, was taken off aspirin, and presented with recurrent low flow alarms 3 days prior to admission.

**Figure 1:**
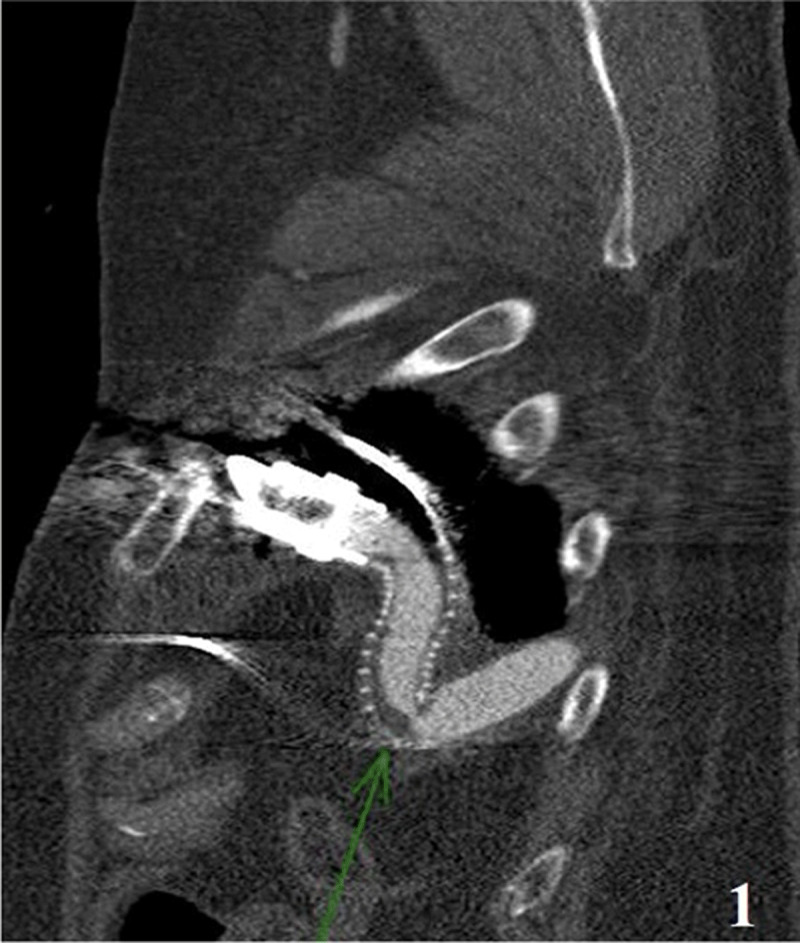
Cardiac computed tomography showing left ventricular assist device outflow graft obstruction.

LVAD interrogation revealed a speed of 5,800 rpm, a flow rate of 3.8 LPM, a pulsatility index (PI) of 2.8, and a power of 4.1 watts. The patient was euvolemic on examination, and his mean arterial pressures (MAPs) were around 80 mm Hg. Continuous cardiac monitoring did not reveal any evidence of arrhythmia. Transthoracic echocardiogram (TTE) showed an optimally functioning LVAD, a well-functioning bioprosthetic mitral valve without evidence of regurgitation, and normal right ventricular size and function. Gated cardiac computed tomography angiography (CCTA) revealed focal narrowing of the LVAD outflow graft caused by focal kinking and an eccentric thrombus (Figure 1).

A multidisciplinary team involving advanced heart failure, interventional cardiology, vascular surgery, and cardiothoracic surgery discussed several treatment options. Due to obesity with a body mass index of 36.6 kg/m^2^, multiple prior surgeries, and lack of social support, the patient was deemed not ready for transplant and considered too high-risk for a pump and graft exchange. Utilizing images obtained from CCTA, a comprehensive assessment of the feasibility and risks associated with LVAD outflow graft intervention was conducted, and it was concluded that LVAD was a reasonable course of action given the patient’s health status.

After successful initiation of anesthesia, the bilateral groins were prepped and draped in the usual sterile fashion. The right common femoral artery was accessed under ultrasound guidance with a micropuncture needle, and a micropuncture wire was advanced followed by micropuncture sheath. A J-wire was advanced into the aorta and a 7F sheath was placed. This was then removed, and two preplaced Perclose ProGlide™ devices (Abbott Vascular) were placed in the right common femoral artery. The patient was systemically heparinized with 10,000 units of heparin, maintaining an activated clotting time > 250 sec. An exchange-length Glidewire Advantage (Terumo International Systems) was then advanced into the ascending aorta, and an 8.8F Terumo Nagare™ sheath was advanced in the proximal descending thoracic aorta.

**Figure 2:**
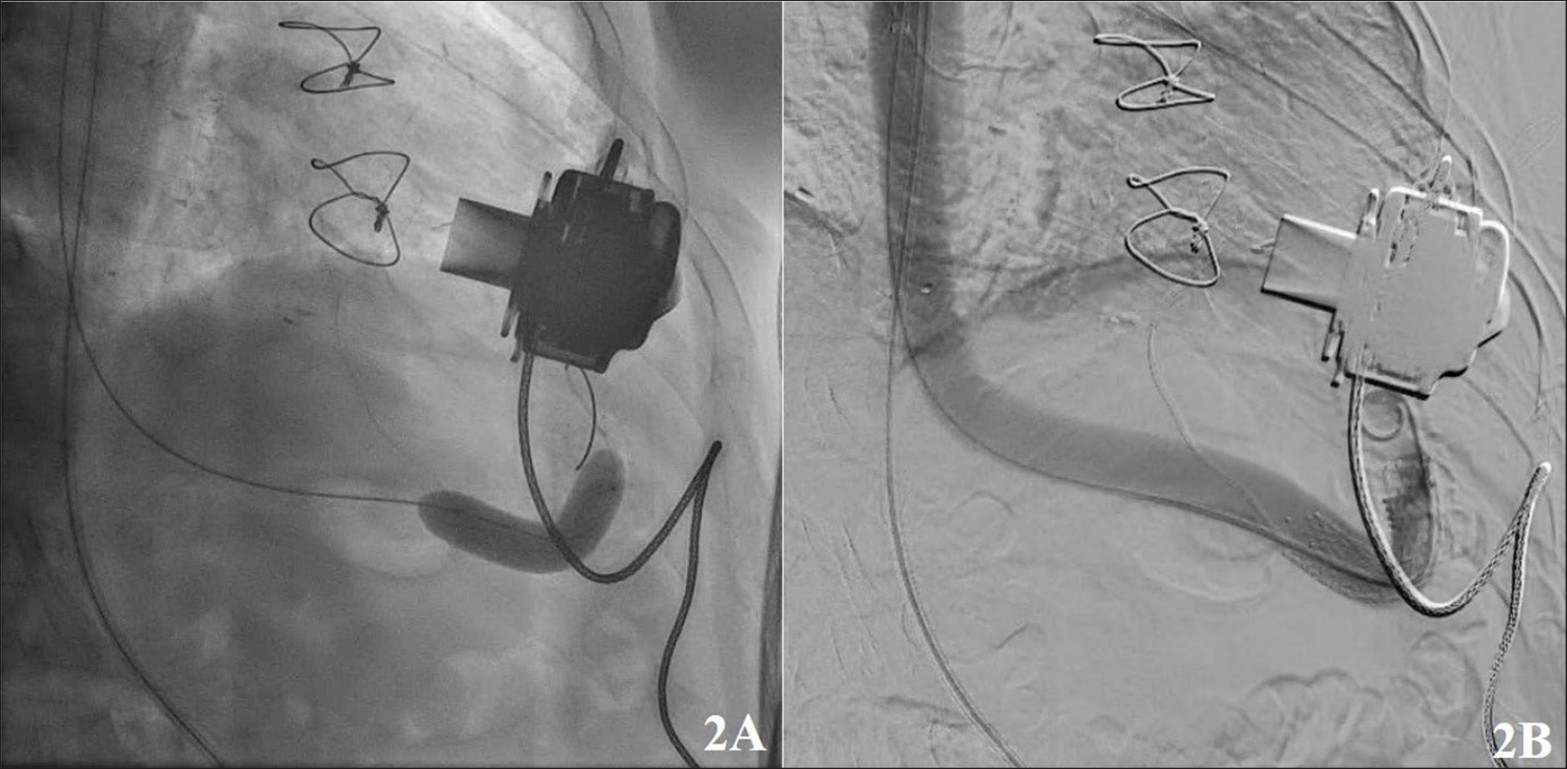
Graft angiogram showing **(A)** balloon dilation of the left ventricular assist device outflow graft obstruction and **(B)** stenting.

Aortography was performed. The steerable sheath was then angulated to engage the orifice of the LVAD outflow limb in the descending thoracic aorta. Using a combination of the exchange-length Glidewire Advantage and a 135 angled Terumo NaviCross catheter, the outflow limb was cannulated and the stenosis was traversed, sustaining access to the proximal LVAD outflow limb near the LVAD motor. Selective angiography was performed. Our wire was exchanged for an exchange-length Lunderquist wire (Cook Medical). The lesion was predilated with a 10 mm × 60 mm Armada 35 catheter (Abbott Cardiovascular) on a 135-cm platform. Following pre-dilatation, a 12 mm × 60 mm EV3 self-expanding bare metal stent (Medtronic) was deployed (Figure 2). The stent was post-dilated to high pressure using a 12 mm × 60 mm Armada 35 balloon. Final angiography revealed a well-apposed stent with no evidence of stenosis.

There was an immediate improvement in the patient’s hemodynamics with no low-flow alarms. After the procedure, LVAD interrogation revealed a speed of 5,900 rpm, a flow rate of 4.9 LPM, a PI of 3.1, and a power of 4.7 watts. Post-procedural CCTA revealed an interval stent placement across the previously seen region of narrowing of the LVAD outflow. There was mild residual narrowing due to focal kinking, which had improved from the prior exam. The patient was discharged home after 2 days on coumadin. Two weeks after hospitalization, follow-up indicated no flow alarms, and the patient remains asymptomatic.

## Discussion

LVADs have revolutionized the management of patients with end-stage heart failure, serving both as a bridge to transplant and as destination therapy.^1^ However, they are not without risk of complications, such as thrombosis, infection, and device hemodynamic or structural malfunction.^2^

This case illustrates the challenges of managing one of the most feared complications associated with LVADs. Our patient presented with recurrent low-flow alarms, raising concerns for the possibility of device obstruction or thrombosis, which can pose significant morbidity and mortality with increased risks of stroke and pump failure.^3^ LVAD thrombosis is a well-recognized complication with a reported incidence of 0.04% to 0.09%.^4^ Pump thrombosis can occur in three different LVAD sites: the inflow cannula, the pump itself (commonly between the impeller and the pump housing), or, less commonly, the outflow graft.^4^ The presentation can vary from hemodynamic instability and hemolysis to something subtle and inconspicuous.^4^

Outflow graft occlusion or stenosis is typically suspected when the device maintains a consistent speed (rpm) but shows a decrease in both power and flow rates. Conversely, an increase in power and flow rates usually indicates a potential internal issue within the device itself.^5^ Although the incidence of pump thrombosis in HeartMate III LVADs is lower than in earlier-generation devices, this remains a major clinical concern that necessitates early and prompt diagnosis.^6^ CCTA is usually an important diagnostic tool providing more data about thrombus location and burden.^7,8^

Open surgical repair remains the primary approach for managing LVAD thrombosis, especially in cases where the etiology is prepump or intrapump.^9,10,11^ Thrombolytics also serve as effective agents for the treatment of prepump or intrapump thrombosis, administered either systemically or through in-device therapy via a transfemoral catheter approach.^12^ However, the literature lacks sufficient data on the efficacy of thrombolytic therapy in outflow graft occlusions.^13^

Percutaneous intervention has been detailed in a limited number of reports and case series and has proven successful to date.^9,10,11^ This approach is particularly valuable due to its feasibility, cost-effectiveness, less invasiveness, and low risk of morbidity, making it beneficial for high-risk surgical patients. A thoughtful and multidisciplinary approach is essential in determining the best course of action for each scenario. In our patient’s case, given his high surgical risk, we believed that catheterization and resolution of the occlusion with stent graft implantation was a viable and effective option. The most challenging aspect of catheter placement in a thrombosed outflow graft is the potential for causing a systemic thromboembolism, particularly cerebrovascular embolisms. Carotid filters or balloon dilatation in the carotid arteries can be employed for cerebral protection, and their successful utilization has been demonstrated.^11,12^

## Conclusion

This case highlights the challenges associated with the management of LVAD outflow graft obstruction. There are limited cases in the literature to establish a standardized treatment approach for outflow graft issues. Embracing a multidisciplinary approach that includes heart failure specialists, interventional cardiologists, vascular surgery, and cardiac surgery is vital. Percutaneous interventions, being fast, feasible, and cost-effective, have consistently shown successful outcomes in all reported cases to date and may be the preferred method for addressing outflow graft occlusions.^11^

## Key Points

- The use of left ventricular assist devices (LVADs) requires recognition of potential thrombosis and obstruction complications and their implications for device malfunction.
- Maximize diagnostic modalities and their importance by understanding the critical role they play, especially transthoracic echocardiography and cardiac computed tomography angiography, in identifying LVAD malfunctions and associated vascular anomalies.
- An integrated management approach is needed to achieve a multidisciplinary approach involving heart failure specialists, interventional cardiologists, and cardiac surgeons to effectively manage LVAD complications and optimize patient outcomes.
